# *N*-(3,5-Dimethyl­phen­yl)-2-methyl­benzamide

**DOI:** 10.1107/S1600536810009116

**Published:** 2010-03-17

**Authors:** B. Thimme Gowda, Miroslav Tokarčík, Jozef Kožíšek, Vinola Zeena Rodrigues, Hartmut Fuess

**Affiliations:** a Department of Chemistry, Mangalore University, Mangalagangotri 574 199, Mangalore, India; b Faculty of Chemical and Food Technology, Slovak Technical University, Radlinského 9, SK-812 37 Bratislava, Slovak Republic; c Institute of Materials Science, Darmstadt University of Technology, Petersenstrasse 23, D-64287 Darmstadt, Germany

## Abstract

In the mol­ecular structure of the title compound, C_16_H_17_NO, the amide group is twisted by 41.8 (2) and 29.0 (2)° out of the planes of the 2-methyl­phenyl and 3,5-dimethyl­phenyl rings, respectively. The two aromatic rings make a dihedral angle of 69.5 (1)°. In the crystal, inter­molecular N—H⋯O hydrogen bonds connect the mol­ecules into *C*(4) chains running along the *c* axis.

## Related literature

For our study of the effect of the substituents on the structures of benzanilides and for related structures, see: Gowda, Foro *et al.* (2008**a* ▶,b* ▶); Gowda, Tokarčík *et al.* (2009 ▶). For synthesis, see: Gowda, Foro *et al.* (2008*b* ▶).
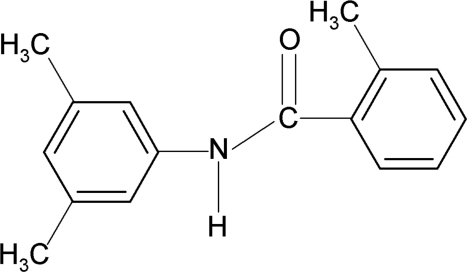


## Experimental

### 

#### Crystal data

C_16_H_17_NO
*M* *_r_* = 239.31
Monoclinic, 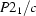

*a* = 10.5174 (5) Å
*b* = 14.9616 (7) Å
*c* = 8.9209 (4) Å
β = 105.373 (4)°
*V* = 1353.54 (11) Å^3^
*Z* = 4
Mo *K*α radiation
μ = 0.07 mm^−1^
*T* = 295 K
0.54 × 0.08 × 0.04 mm

#### Data collection

Oxford Diffraction Xcalibur Ruby Gemini diffractometer
Absorption correction: multi-scan (*CrysAlis PRO*; Oxford Diffraction, 2009 ▶) *T*_min_ = 0.957, *T*_max_ = 0.992
14671 measured reflections
2548 independent reflections
1628 reflections with *I* > 2σ(*I*)
*R*_int_ = 0.041

#### Refinement

*R*[*F* ^2^ > 2σ(*F* ^2^)] = 0.037
*wR*(*F* ^2^) = 0.120
*S* = 0.97
2548 reflections
170 parameters
H-atom parameters constrained
Δρ_max_ = 0.13 e Å^−3^
Δρ_min_ = −0.12 e Å^−3^

### 

Data collection: *CrysAlis PRO* (Oxford Diffraction, 2009 ▶); cell refinement: *CrysAlis PRO*; data reduction: *CrysAlis PRO*; program(s) used to solve structure: *SHELXS97* (Sheldrick, 2008 ▶); program(s) used to refine structure: *SHELXL97* (Sheldrick, 2008 ▶); molecular graphics: *ORTEP-3* (Farrugia, 1997 ▶) and *DIAMOND* (Brandenburg, 2002 ▶); software used to prepare material for publication: *SHELXL97*, *PLATON* (Spek, 2009 ▶) and *WinGX* (Farrugia, 1999 ▶).

## Supplementary Material

Crystal structure: contains datablocks I, global. DOI: 10.1107/S1600536810009116/tk2640sup1.cif

Structure factors: contains datablocks I. DOI: 10.1107/S1600536810009116/tk2640Isup2.hkl

Additional supplementary materials:  crystallographic information; 3D view; checkCIF report

## Figures and Tables

**Table 1:** Hydrogen-bond geometry (Å, °)

*D*—H⋯*A*	*D*—H	H⋯*A*	*D*⋯*A*	*D*—H⋯*A*
N1—H1*N*⋯O1^i^	0.86	2.06	2.8935 (16)	163

Symmetry code: (i) 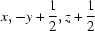.

**Fig. 1.**
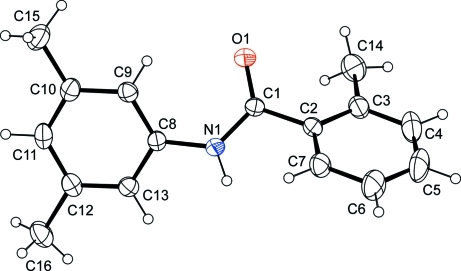
Molecular structure of (I) showing the atom labelling scheme. Displacement ellipsoids are drawn at the 30% probability level and H atoms are represented as small spheres of arbitrary radii.

**Fig. 2.**
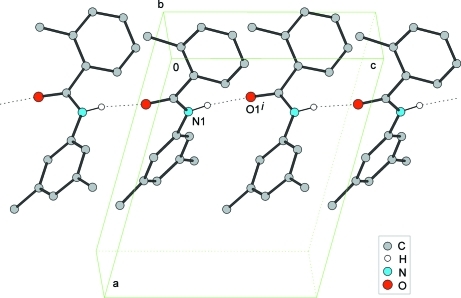
Supramolecular chain formation in (I) with hydrogen bonds shown as dashed lines. Symmetry code (i): x, -y+1/2, z+1/2. H atoms not involved in hydrogen bonding were omitted for reasons of clarity.

## supplementary crystallographic information

### Comment

As a part of our efforts to explore the effect of the substituents on the structures of benzanilides (Gowda, Foro *et al.*, 2008*a, b*, Gowda, Tokarčík *et al.*, 2009), in the present work, the structure of 2-methyl-*N*-(3,5-dimethylphenyl)benzamide (I) has been determined. In the structure of (I) (Fig. 1), the N—H and C=O groups are in an antiperiplanar conformation. This conformation is similar to those observed, *e.g.* in 2-methyl-*N*-(phenyl)benzamide (II) (Gowda, Foro *et al.*, 2008*a*), 2-methyl-*N*-(2,6-dimethylphenyl)benzamide (III) (Gowda, Foro *et al.*, 2008*b*), and 2-methyl-*N*-(2,4-dimethylphenyl)benzamide (IV) (Gowda, Tokarčík *et al.*, 2009). Further, in (I) the conformation of the C=O group to the methyl substituent in the 2-methylphenyl ring is *syn*. This conformation is similar to those observed in (II) and (IV). The bond parameters in (I) are similar to those in (II), (III), (IV), and other benzanilides.

In the molecule, the amido group is twisted 41.8 (2)° and 29.0 (2)° out of the planes of the 2-methylphenyl and the 3,5-dimethylphenyl rings, respectively. The two aromatic rings make the dihedral angle of 69.5 (1)°. Intermolecular N–H···O hydrogen bonds (Table 1) connect the molecules into chains running along the *c*-axis (Fig. 2).

### Experimental

Compound (I) as prepared according to the method described by Gowda, Foro *et al.* (2008*b*). Colourless blocks of (I) were obtained by slow evaporation from an ethanol solution (0.5 g in about 25 ml of ethanol) held at room temperature.

### Refinement

All hydrogen atoms were positioned with idealized geometry using a riding model with C–H = 0.93-0.96 Å and N–H = 0.86 Å. The *U*_iso_(H) values were set at 1.2*U*_eq_(C-aromatic, N) and 1.5*U*_eq_(*C*-methyl). The methyl groups with the carbon atoms C14, C15 and C16 exhibit orientational disorder in the positions of their H atoms, and each group was modelled by two sets of methyl hydrogen atoms. The refined occupancies are 0.82 (3) and 0.18 (3) for the C14-mehtyl group, 0.60 (3) and 0.40 (3) for the C15 group, 0.73 (2) and 0.27 (2) for the C16 group.

### Crystal data

**Table:**

C_16_H_17_NO	*F*(000) = 512
*M**_r_* = 239.31	*D*_x_ = 1.174 Mg m^−^^3^
Monoclinic, *P*2_1_/*c*	Mo *K*α radiation, λ = 0.71073 Å
Hall symbol: -P 2ybc	Cell parameters from 5268 reflections
*a* = 10.5174 (5) Å	θ = 2.0–29.4°
*b* = 14.9616 (7) Å	µ = 0.07 mm^−^^1^
*c* = 8.9209 (4) Å	*T* = 295 K
β = 105.373 (4)°	Needle, colourless
*V* = 1353.54 (11) Å^3^	0.54 × 0.08 × 0.04 mm
*Z* = 4	

### Data collection

**Table:**

Oxford Diffraction Xcalibur Ruby Gemini diffractometer	2548 independent reflections
graphite	1628 reflections with *I* > 2σ(*I*)
Detector resolution: 10.434 pixels mm^-1^	*R*_int_ = 0.041
ω scans	θ_max_ = 25.6°, θ_min_ = 2.0°
Absorption correction: multi-scan (*CrysAlis PRO*; Oxford Diffraction, 2009)	*h* = −12→12
*T*_min_ = 0.957, *T*_max_ = 0.992	*k* = −18→18
14671 measured reflections	*l* = −10→10

### Refinement

**Table:**

Refinement on *F*^2^	Primary atom site location: structure-invariant direct methods
Least-squares matrix: full	Secondary atom site location: difference Fourier map
*R*[*F*^2^ > 2σ(*F*^2^)] = 0.037	Hydrogen site location: inferred from neighbouring sites
*wR*(*F*^2^) = 0.120	H-atom parameters constrained
*S* = 0.97	[exp(2.50(sinθ/λ)^2^)]/[σ^2^(*F*_o_^2^) + (0.094*P*)^2^], where *P* = 0.33333*F*_o_^2^ + 0.66667*F*_c_^2^
2548 reflections	(Δ/σ)_max_ < 0.001
170 parameters	Δρ_max_ = 0.13 e Å^−^^3^
0 restraints	Δρ_min_ = −0.12 e Å^−^^3^

### Special details

**Table:**

Geometry. All esds (except the esd in the dihedral angle between two l.s. planes) are estimated using the full covariance matrix. The cell esds are taken into account individually in the estimation of esds in distances, angles and torsion angles; correlations between esds in cell parameters are only used when they are defined by crystal symmetry. An approximate (isotropic) treatment of cell esds is used for estimating esds involving l.s. planes.
Refinement. Refinement of *F*^2^ against ALL reflections. The weighted *R*-factor wR and goodness of fit *S* are based on *F*^2^, conventional *R*-factors *R* are based on *F*, with *F* set to zero for negative *F*^2^. The threshold expression of *F*^2^ > σ(*F*^2^) is used only for calculating *R*-factors(gt) etc. and is not relevant to the choice of reflections for refinement. *R*-factors based on *F*^2^ are statistically about twice as large as those based on *F*, and *R*- factors based on ALL data will be even larger.

### Fractional atomic coordinates and isotropic or equivalent isotropic displacement parameters (Å^2^)

**Table:**

	*x*	*y*	*z*	*U*_iso_*/*U*_eq_	Occ. (<1)
C1	0.20367 (15)	0.19330 (10)	0.08175 (15)	0.0348 (4)	
C2	0.09976 (16)	0.14838 (10)	0.14205 (17)	0.0392 (4)	
C3	−0.02376 (17)	0.12792 (12)	0.0426 (2)	0.0503 (5)	
C4	−0.1141 (2)	0.08515 (16)	0.1073 (3)	0.0724 (6)	
H4	−0.1967	0.0705	0.0435	0.087*	
C5	−0.0850 (2)	0.06389 (17)	0.2623 (3)	0.0835 (7)	
H5	−0.148	0.0359	0.3021	0.1*	
C6	0.0366 (2)	0.08381 (16)	0.3589 (3)	0.0760 (7)	
H6	0.0564	0.0695	0.464	0.091*	
C7	0.1290 (2)	0.12525 (12)	0.29829 (19)	0.0527 (5)	
H7	0.2121	0.1379	0.3629	0.063*	
C8	0.38098 (14)	0.30756 (10)	0.15513 (15)	0.0354 (4)	
C9	0.46745 (15)	0.27564 (11)	0.07407 (16)	0.0396 (4)	
H9	0.4536	0.2198	0.0266	0.048*	
C10	0.57512 (15)	0.32734 (12)	0.06388 (17)	0.0454 (4)	
C11	0.59283 (16)	0.41058 (12)	0.13406 (18)	0.0489 (4)	
H11	0.6637	0.4456	0.1256	0.059*	
C12	0.50798 (17)	0.44351 (11)	0.21670 (17)	0.0449 (4)	
C13	0.40205 (16)	0.39107 (11)	0.22612 (16)	0.0398 (4)	
H13	0.344	0.4119	0.2807	0.048*	
C14	−0.0633 (2)	0.15265 (17)	−0.1269 (2)	0.0743 (6)	
H14A	−0.1568	0.1457	−0.1673	0.112*	0.82 (3)
H14B	−0.0187	0.1144	−0.1829	0.112*	0.82 (3)
H14C	−0.0395	0.2137	−0.1386	0.112*	0.82 (3)
H14D	0.0135	0.1703	−0.1585	0.112*	0.18 (3)
H14E	−0.1248	0.2014	−0.143	0.112*	0.18 (3)
H14F	−0.1037	0.1021	−0.1872	0.112*	0.18 (3)
C15	0.67092 (19)	0.29122 (17)	−0.0193 (2)	0.0651 (6)	
H15A	0.7143	0.34	−0.0554	0.098*	0.60 (3)
H15B	0.6244	0.2558	−0.1063	0.098*	0.60 (3)
H15C	0.7353	0.2548	0.0507	0.098*	0.60 (3)
H15D	0.6684	0.2271	−0.0186	0.098*	0.40 (3)
H15E	0.7583	0.3112	0.0324	0.098*	0.40 (3)
H15F	0.6474	0.3123	−0.1247	0.098*	0.40 (3)
C16	0.5318 (2)	0.53329 (13)	0.2949 (2)	0.0644 (5)	
H16A	0.5572	0.5754	0.227	0.097*	0.73 (2)
H16B	0.6009	0.5283	0.3896	0.097*	0.73 (2)
H16C	0.4525	0.5535	0.3182	0.097*	0.73 (2)
H16D	0.5166	0.5294	0.3962	0.097*	0.27 (2)
H16E	0.4728	0.5765	0.2336	0.097*	0.27 (2)
H16F	0.6213	0.5513	0.305	0.097*	0.27 (2)
N1	0.27245 (13)	0.25733 (9)	0.17550 (13)	0.0399 (3)	
H1N	0.2474	0.2691	0.2576	0.048*	
O1	0.22394 (11)	0.17203 (8)	−0.04280 (11)	0.0456 (3)	

### Atomic displacement parameters (Å^2^)

**Table:**

	*U*^11^	*U*^22^	*U*^33^	*U*^12^	*U*^13^	*U*^23^
C1	0.0372 (8)	0.0346 (8)	0.0317 (7)	0.0014 (7)	0.0078 (6)	0.0008 (6)
C2	0.0428 (9)	0.0315 (8)	0.0444 (8)	−0.0029 (7)	0.0137 (7)	−0.0046 (6)
C3	0.0426 (10)	0.0445 (10)	0.0629 (11)	−0.0037 (8)	0.0122 (8)	−0.0086 (8)
C4	0.0485 (11)	0.0649 (14)	0.1019 (16)	−0.0159 (10)	0.0167 (11)	−0.0023 (12)
C5	0.0744 (16)	0.0734 (16)	0.1135 (19)	−0.0260 (13)	0.0438 (15)	0.0144 (13)
C6	0.0901 (17)	0.0724 (15)	0.0719 (12)	−0.0242 (13)	0.0327 (12)	0.0169 (11)
C7	0.0602 (11)	0.0481 (10)	0.0506 (10)	−0.0128 (8)	0.0163 (8)	0.0044 (8)
C8	0.0360 (8)	0.0395 (9)	0.0290 (7)	−0.0040 (7)	0.0056 (6)	0.0048 (6)
C9	0.0396 (9)	0.0428 (9)	0.0341 (7)	−0.0008 (7)	0.0055 (6)	0.0004 (6)
C10	0.0359 (9)	0.0604 (11)	0.0377 (8)	−0.0007 (8)	0.0058 (6)	0.0074 (7)
C11	0.0387 (9)	0.0580 (11)	0.0463 (8)	−0.0133 (8)	0.0050 (7)	0.0113 (8)
C12	0.0486 (10)	0.0413 (9)	0.0399 (8)	−0.0071 (8)	0.0031 (7)	0.0065 (7)
C13	0.0444 (9)	0.0393 (9)	0.0354 (7)	−0.0013 (7)	0.0101 (6)	0.0020 (6)
C14	0.0483 (11)	0.1004 (18)	0.0638 (12)	−0.0075 (12)	−0.0037 (9)	−0.0105 (11)
C15	0.0441 (10)	0.0897 (16)	0.0651 (11)	0.0000 (10)	0.0209 (9)	−0.0018 (10)
C16	0.0745 (14)	0.0471 (11)	0.0683 (11)	−0.0196 (10)	0.0134 (10)	−0.0037 (9)
N1	0.0461 (8)	0.0433 (8)	0.0341 (6)	−0.0087 (6)	0.0173 (5)	−0.0051 (5)
O1	0.0505 (7)	0.0517 (7)	0.0363 (6)	−0.0048 (6)	0.0148 (5)	−0.0068 (5)

### Geometric parameters (Å, °)

**Table:**

C1—O1	1.2276 (17)	C12—C13	1.383 (2)
C1—N1	1.3490 (19)	C12—C16	1.504 (2)
C1—C2	1.499 (2)	C13—H13	0.93
C2—C7	1.389 (2)	C14—H14A	0.96
C2—C3	1.399 (2)	C14—H14B	0.96
C3—C4	1.391 (3)	C14—H14C	0.96
C3—C14	1.504 (3)	C14—H14D	0.96
C4—C5	1.372 (3)	C14—H14E	0.96
C4—H4	0.93	C14—H14F	0.96
C5—C6	1.372 (3)	C15—H15A	0.96
C5—H5	0.93	C15—H15B	0.96
C6—C7	1.378 (3)	C15—H15C	0.96
C6—H6	0.93	C15—H15D	0.96
C7—H7	0.93	C15—H15E	0.96
C8—C9	1.388 (2)	C15—H15F	0.96
C8—C13	1.392 (2)	C16—H16A	0.96
C8—N1	1.4180 (19)	C16—H16B	0.96
C9—C10	1.394 (2)	C16—H16C	0.96
C9—H9	0.93	C16—H16D	0.96
C10—C11	1.384 (2)	C16—H16E	0.96
C10—C15	1.501 (3)	C16—H16F	0.96
C11—C12	1.390 (2)	N1—H1N	0.86
C11—H11	0.93		
			
O1—C1—N1	123.50 (13)	C11—C12—C16	120.75 (16)
O1—C1—C2	121.78 (13)	C12—C13—C8	121.01 (15)
N1—C1—C2	114.72 (12)	C12—C13—H13	119.5
C7—C2—C3	120.21 (16)	C8—C13—H13	119.5
C7—C2—C1	118.90 (15)	C3—C14—H14A	109.5
C3—C2—C1	120.87 (14)	C3—C14—H14B	109.5
C4—C3—C2	117.28 (17)	C3—C14—H14C	109.5
C4—C3—C14	119.57 (18)	C3—C14—H14D	109.5
C2—C3—C14	123.12 (16)	C3—C14—H14E	109.5
C5—C4—C3	122.1 (2)	H14D—C14—H14E	109.5
C5—C4—H4	119	C3—C14—H14F	109.5
C3—C4—H4	119	H14D—C14—H14F	109.5
C4—C5—C6	120.27 (19)	H14E—C14—H14F	109.5
C4—C5—H5	119.9	C10—C15—H15A	109.5
C6—C5—H5	119.9	C10—C15—H15B	109.5
C5—C6—C7	119.2 (2)	C10—C15—H15C	109.5
C5—C6—H6	120.4	C10—C15—H15D	109.5
C7—C6—H6	120.4	C10—C15—H15E	109.5
C6—C7—C2	120.94 (19)	H15D—C15—H15E	109.5
C6—C7—H7	119.5	C10—C15—H15F	109.5
C2—C7—H7	119.5	H15D—C15—H15F	109.5
C9—C8—C13	119.91 (14)	H15E—C15—H15F	109.5
C9—C8—N1	123.04 (14)	C12—C16—H16A	109.5
C13—C8—N1	117.00 (13)	C12—C16—H16B	109.5
C8—C9—C10	119.92 (15)	C12—C16—H16C	109.5
C8—C9—H9	120	C12—C16—H16D	109.5
C10—C9—H9	120	C12—C16—H16E	109.5
C11—C10—C9	118.94 (15)	H16D—C16—H16E	109.5
C11—C10—C15	121.33 (16)	C12—C16—H16F	109.5
C9—C10—C15	119.71 (17)	H16D—C16—H16F	109.5
C10—C11—C12	122.04 (15)	H16E—C16—H16F	109.5
C10—C11—H11	119	C1—N1—C8	127.92 (12)
C12—C11—H11	119	C1—N1—H1N	116
C13—C12—C11	118.16 (15)	C8—N1—H1N	116
C13—C12—C16	121.09 (16)		
			
O1—C1—C2—C7	136.94 (16)	N1—C8—C9—C10	−177.47 (13)
N1—C1—C2—C7	−42.4 (2)	C8—C9—C10—C11	−0.7 (2)
O1—C1—C2—C3	−41.5 (2)	C8—C9—C10—C15	177.92 (15)
N1—C1—C2—C3	139.08 (15)	C9—C10—C11—C12	1.2 (2)
C7—C2—C3—C4	0.7 (3)	C15—C10—C11—C12	−177.41 (16)
C1—C2—C3—C4	179.16 (17)	C10—C11—C12—C13	−0.9 (2)
C7—C2—C3—C14	178.64 (19)	C10—C11—C12—C16	178.48 (16)
C1—C2—C3—C14	−2.9 (3)	C11—C12—C13—C8	0.1 (2)
C2—C3—C4—C5	0.4 (3)	C16—C12—C13—C8	−179.26 (15)
C14—C3—C4—C5	−177.6 (2)	C9—C8—C13—C12	0.3 (2)
C3—C4—C5—C6	−0.7 (4)	N1—C8—C13—C12	177.91 (13)
C4—C5—C6—C7	−0.1 (4)	O1—C1—N1—C8	−3.4 (2)
C5—C6—C7—C2	1.2 (3)	C2—C1—N1—C8	176.00 (14)
C3—C2—C7—C6	−1.5 (3)	C9—C8—N1—C1	−27.9 (2)
C1—C2—C7—C6	−179.97 (18)	C13—C8—N1—C1	154.64 (15)
C13—C8—C9—C10	0.0 (2)		

### Hydrogen-bond geometry (Å, °)

**Table:**

*D*—H···*A*	*D*—H	H···*A*	*D*···*A*	*D*—H···*A*
N1—H1N···O1^i^	0.86	2.06	2.8935 (16)	163

Symmetry codes: (i) *x*, −*y*+1/2, *z*+1/2.
